# Microstructure Evolution and Enhanced Hot Workability of TiC/Ti-6Al-4V Composites Fabricated by Melt Hydrogenation

**DOI:** 10.3390/ma15248884

**Published:** 2022-12-12

**Authors:** Xuan Wang, Siyu Chen, Yingmei Tan, Longhui Yao, Liang Wang, Yanqing Su, Jingjie Guo

**Affiliations:** 1 School of Materials Science and Engineering, Nanling Campus, Jilin University, No. 5988 Renmin Street, Changchun 130025, China; 2 National Key Laboratory for Precision Hot Processing of Metals, Harbin Institute of Technology, Harbin 150001, China

**Keywords:** Ti-6Al-4V composites, melt hydrogenation, hot workability, TiC phase, dynamic recrystallization

## Abstract

Improving the hot workability and reducing the processing cost are critical steps to expanding the application of TiC/Ti-6Al-4V composites. This study employed melt hydrogenation to fabricate TiC/Ti-6Al-4V composites under a mixed atmosphere of hydrogen and argon. Experimental results indicated that hydrogen had an obvious influence on the growth and morphology of eutectic TiC particles, and the size of eutectic TiC and primary β grains was significantly increased. As a result, large-sized eutectic TiC was distributed along the grain boundaries of primary β grains. Hot compression results showed that the peak flowing stress of composites was reduced by hydrogen, which resulted in an improvement of hot workability, especially in the (α + β) phase region, and the best results were obtained at 900 °C/0.01 s^−1^, at which the peak stress decreased from 241 ± 9 to 190 ± 8 MPa (a decrease of 21.2%). Inspection of the microstructure after hot compression showed that hydrogen improved the proportion of DRX grains from ~62.7% to ~83.2%, and hydrogen also decreased the density of dislocations, which were attributed to hydrogen accelerating atomic diffusion. Enhanced hot workability resulted from hydrogen atoms decreasing the atomic bonding force of the titanium matrix, hydrogen reducing the β/(α + β) transition temperature, the higher proportion of DRX, and the higher mobility of dislocations. It is expected that the findings of this study may support the development of a simple and efficient method to reduce the processing cost of TiC/Ti-6Al-4V composites.

## 1. Introduction

Titanium matrix composites (TMCs) have high specific strength and excellent high-temperature performance, meaning they have wide application prospects in aerospace fields [1,2,3], including the development of many key structural components such as aircraft fuselage skin, wings, support beams, engine blades, and compressors. In recent years, in situ fabricating has resulted in a simple manufacturing process, lower costs, and good interface bonding in as-cast TMCs. It is considered a promising manufacturing method for TMCs [4]. TiC has excellent properties across the board, including good compatibility and stability with the titanium matrix [5], meaning it is regarded as one of the suitable reinforcements for TMCs. Wei et al. [6] reported that Ti-6Al-4V (Ti64) matrix composites with 5 vol.% TiC addition had excellent mechanical properties, and their yield strength and ultimate strength at room temperature were 900.7 and 1005.2 MPa, respectively. However, the high processing cost is still the main issue limiting the wide application of TiC/Ti64 composites.

Ti64 matrix alloy has high deforming resistance, and the addition of TiC particles will further increase the hot processing resistance [7,8]. Therefore, improving the hot workability is a critical step to expanding the applications of TiC/Ti64 composites. Hydrogenation technology [9] treats hydrogen as a temporary alloying element, which can significantly decrease the flowing stress and then improve the hot workability of TMCs. The dissolution of hydrogen atoms (or reaction with Ti atoms to form hydrides) is reversible, meaning hydrogen atoms can easily be removed by vacuum annealing. After vacuum annealing, the vast majority of hydrogen atoms are extracted from the materials, which ensures the low content of hydrogen atoms in titanium-based alloys used at room temperature. Based on traditional hydrogenation, this study presents a more advanced melt hydrogenation technology [10,11], which involves melting the materials directly within a mixed atmosphere of hydrogen and argon. Wang et al. [12] reported that melt hydrogenation could allow TMCs to be deformed at lower temperatures and higher strain rates. Lin et al. [13], meanwhile, reported that melt hydrogenation decreased the peak flowing stress of TMCs from 240 MPa to 199 MPa at 850 °C/0.01 s^−1^. Therefore, melt hydrogenation seems a feasible method to improve the hot workability of TMCs.

As for TiC-reinforced Ti-6Al-4V composites, TiC has different morphologies, including primary TiC and eutectic TiC [14]. It has been reported that melt hydrogenation could change the morphology of ceramic particles by accelerating atomic diffusion [15], but its effect on the growth and morphology of TiC has not yet been reported. Therefore, this study takes a Ti-6Al-4V alloy reinforced with 2.5 vol.% TiC as the research object and investigates the effect of melt hydrogenation on the morphology and distribution of the TiC phase, hot compression behaviors, and the mechanism for improvement of hot workability. The findings of this study will support the development of a simple and efficient method to reduce the processing cost of TiC/Ti-6Al-4V composites.

## 2. Materials and Methods

The materials used in this study were TiC/Ti-6Al-4V composites, and the volume fraction of TiC particles was 2.5%. Raw materials were sponge titanium, graphite powder, Al-58.18 wt.% V master alloy, and pure aluminum. All raw materials had high purity (99.9%). After the in situ reaction of Ti + C → TiC during melting, the TiC particles were fabricated into composites by adding graphite powder. Melt hydrogenation was conducted in an arc melting furnace, and the hydrogen percentages in mixed gas were 0 and 20%. In order to guarantee the chemical homogeneity of as-cast composites, all the materials were melted five times, and hydrogen atoms were dissolved in the melt during melting processing. The hydrogen content in as-cast composites was measured by a hydrogen–oxygen analyzer (LECO ONH836, St. Joseph, MO, USA), where the mass of samples for hydrogen content measurement was 0.15 g. Another two parallel samples for hydrogen content measurement were also tested to ensure good repeatability. The β/(α + β) phase transition temperatures of as-cast composites with and without hydrogen were tested by differential scanning calorimetry (DSC), and the DSC tests were repeated three times to ensure repeatability. The size of samples for DSC analysis was Φ 3 × 3.5 mm, and the range of temperature measurement was 25 to 1200 °C with the same heating and cooling rate of 20 °C/min. The hydrogen content and β/(α + β) transition temperature of TiC/Ti64 composites are shown in Table 1.

The microstructure of as-cast composites was observed by scanning electron microscopy (SEM, FEI Quanta 200F, Hillsboro, OR, USA), and samples for SEM observation were etched with a solution of 10% HF, 10% HNO_3_, and 80% H_2_O for 10, 30, and 60 s, respectively. The length of TiC particles was measured by Nano Measurer 2.0, and at least 50 samples were collected, then the average value was deduced. The size of samples for hot compression was Φ 6 × 9 mm, and hot compression was conducted using a Gleeble-1500D dynamic thermal simulation (Gleeble, Rensselaer County, NY, USA) machine with a total compression strain of 50%. All samples for hot compression were covered with silica gel to avoid the escape of hydrogen atoms during hot compression, and each condition was repeated three times per sample to ensure good repeatability. The heating rate for hot compression was 10 °C/s, and the holding time at the evaluated temperature was 3 min. The hot compression temperatures were 700, 800, and 900 °C, and the strain rates were 0.01 and 0.001 s^−1^. After hot compression, the samples were observed by electron back-scatted diffraction (EBSD, FEI Quanta 200F, Hillsboro, OR, USA) and transmission electron microscopy (TEM, FEI Talos F200X, Hillsboro, OR, USA). The solution for the preparation of EBSD and TEM samples was per-chloric acid (6%), n-butyl alcohol (34%), and methanol (60%).

## 3. Results

**Figure 1:**
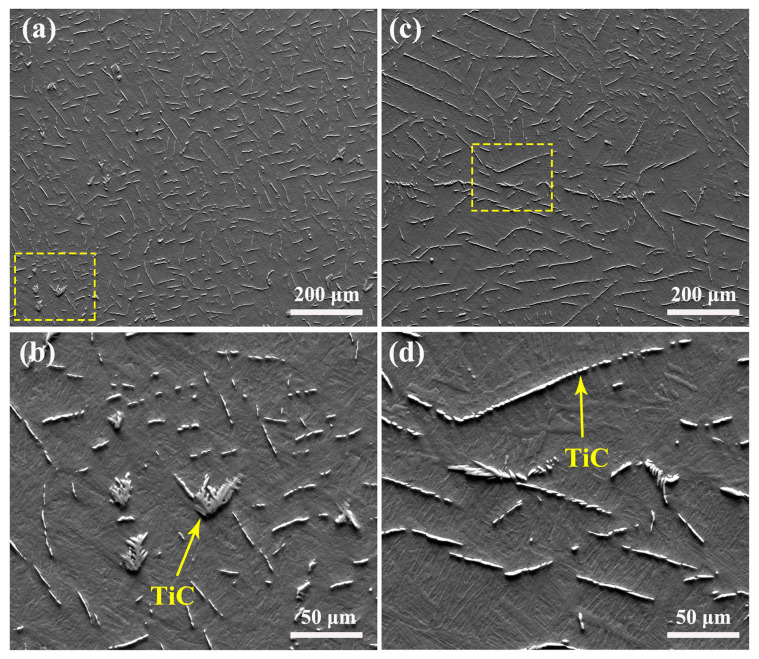
As-cast microstructure of TiC/Ti64 composites etching for 10 s: (**a**,**b**) without melt hydrogenation, (**c**,**d**) with melt hydrogenation.

The as-cast microstructures of TiC/Ti64 composites without and with melt hydrogenation are shown in Figure 1. According to the results shown in Figure 1a, the TiC particles have a homogeneous distribution in TiC/Ti64 composites without hydrogenation, and most TiC particles have a shot-rod morphology. The enlarged image shown in Figure 1b indicates that some TiC particles have a dendritic morphology (marked by the yellow arrow), and the average length of TiC particles is measured to be ~35 ± 8 μm. The distribution of TiC particles in TiC/Ti64 composites after melt hydrogenation is shown in Figure 1c; it can be seen that many TiC particles are longer and the primary β grain boundaries (particle-free zones) are more obvious compared to those without hydrogenation. The enlarged images shown in Figure 1d indicate that the TiC particles have a similar morphology to those shown in Figure 1b but with a greater length, measured to be ~72 ± 11 μm on average. Overall, the results shown in Figure 1 indicate that melt hydrogenation can significantly change the morphology and distribution of TiC particles. After hydrogenation, longer TiC particles are prone to concentrating at the primary β grain boundaries.

**Figure 2:**
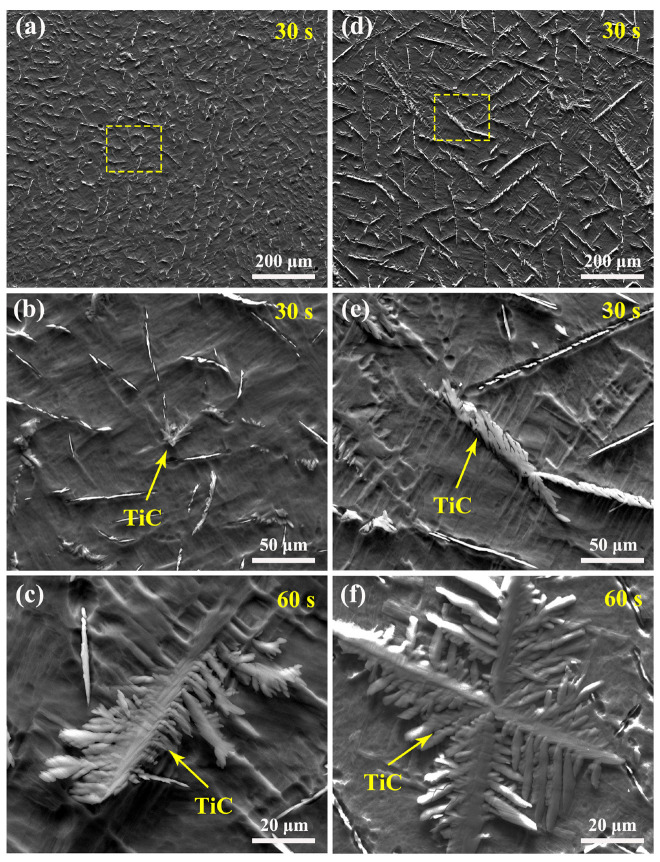
As-cast microstructure of TiC/Ti64 composites with deep etch: (**a**–**c**) without melt hydrogenation, (**d**–**f**) with melt hydrogenation.

Figure 2 shows the as-cast microstructures of TiC/Ti64 composites with longer etching times (30 and 60 s). As shown in Figure 2a,b, the TiC particles in composites without hydrogenation have a homogeneous distribution and shorter length, and the microstructure with a longer etching time indicates that the TiC particles have a feather-like morphology. After further increasing the etching time (60 s), the enlarged image shown in Figure 2c indicates that the TiC has a feather-like or dendritic morphology, and the secondary small dendritic arms can be seen clearly. With melt hydrogenation, the TiC particles with longer lengths distribute along the primary β grain boundaries (Figure 2d), and the enlarged image shown in Figure 2e indicates the TiC in hydrogenated composites also has a feather-like morphology. With a longer etching time (60 s, Figure 2f), the TiC has a typical snow-like or dendritic morphology, where the secondary dendritic arms’ spacing is significantly wider than that shown in Figure 2c, and the length of secondary dendritic arms is also longer. The results shown in Figure 2 indicate that melt hydrogenation has an obvious influence on the growth or morphology of TiC particles.

**Figure 3:**
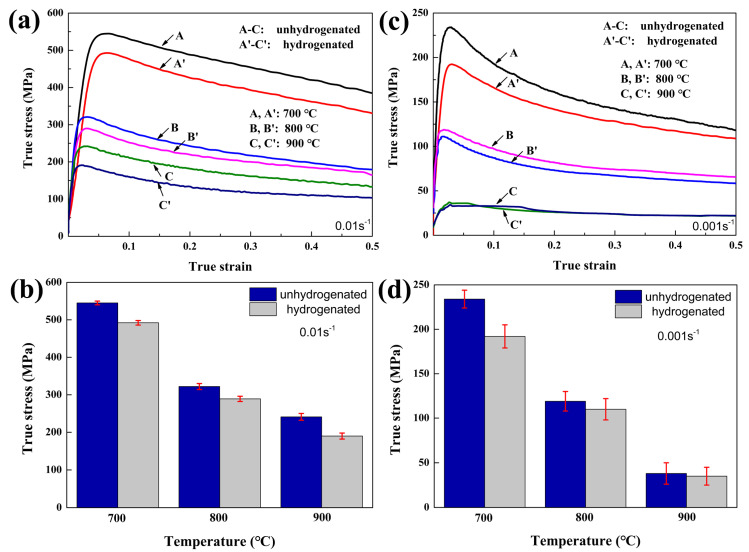
True strain-stress curves and peak flowing stress of TiC/Ti64 composites: (**a**,**b**) without melt hydrogenation, strain rate of 0.01 s^−1^; (**c**,**d**) with melt hydrogenation, strain rate of 0.001 s^−1^.

True hot compressing strain–stress curves of TiC/Ti64 composites with different hydrogen contents are shown in Figure 3, in which the compressing temperatures are 700, 800, and 900 °C, and the strain rates are 0.01 and 0.001 s^−1^, respectively. As shown in Figure 3a, all the curves reach the peak point after hot compression and then drop steadily with increasing strain, which indicates a typical dynamic recrystallization (DRX) characteristic. According to the statistical results shown in Figure 3b, when the stain rate is 0.01 s^−1^, melt hydrogenation decreases the peak flowing stress by ~53 MPa (from 545 ± 5 to 492 ± 6 MPa), ~33 MPa (from 322 ± 8 to 289 ± 7 MPa), and ~51 MPa (from 241 ± 9 to 190 ± 8 MPa) at 700, 800, and 900 °C, respectively. Similarly, when the stain rate is 0.001 s^−1^ (Figure 3c,d), melt hydrogenation decreases the peak flowing stress by ~42 MPa (from 234 ± 10 to 192 ± 13 MPa), ~9 MPa (from 119 ± 11 to 110 ± 12 MPa), and ~3 MPa (from 38 ± 9 to 35 ± 8 MPa) at 700, 800, and 900 °C, respectively. It can be seen that the melt hydrogenation-induced reduction in peak flowing stress is most obvious with a strain rate of 0.01 s^−1^, and the best results can be obtained when compressed at 900 °C/0.01 s^−1^. The hot compression results indicate that melt hydrogenation can decrease the flowing stress of TiC/Ti64 composites and then improve their hot workability effectively when deformed in the (α + β) phase region.

**Figure 4:**
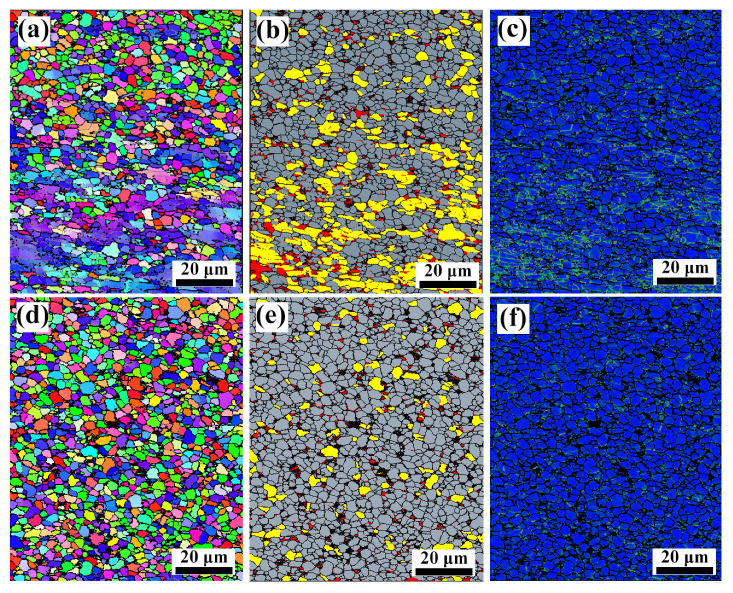
IPF, DRX, and KAM maps of TiC/Ti64 composites deformed at 900 °C/0.001 s^−1^: (**a**–**c**) without melt hydrogenation, (**d**–**f**) with melt hydrogenation.

Figure 4 shows inverse pole figures (IPF), dynamic recrystallization (DRX) maps, and kernel average misorientation (KAM) maps of TiC/Ti64 composites after deforming at 900 °C and a strain rate of 0.001 s^−1^. As shown in Figure 4a, the TiC/Ti64 composites without hydrogenation have dual grain structures: strip-like grains and equiaxial grains. The DRX map shown in Figure 4b further confirms that the strip-like grains are deformed grains, the equiaxial grains are DRX grains, and the proportion of DRX grains is ~62.7%. The KAM map shown in Figure 4c indicates that there is a higher density of dislocations in deformed gains and a lower density of dislocations in DRX grains. After melt hydrogenation, the TiC/Ti64 composites have fully equiaxial grains after hot deformation, as shown in Figure 4d. The DRX has been effectively accelerated, and the proportion of DRX grains in TiC/Ti64 composites after melt hydrogenation is ~83.2%, as shown in Figure 4e. The KAM map shown in Figure 4f also indicates that melt hydrogenation can significantly decrease the density of dislocations.

**Figure 5:**
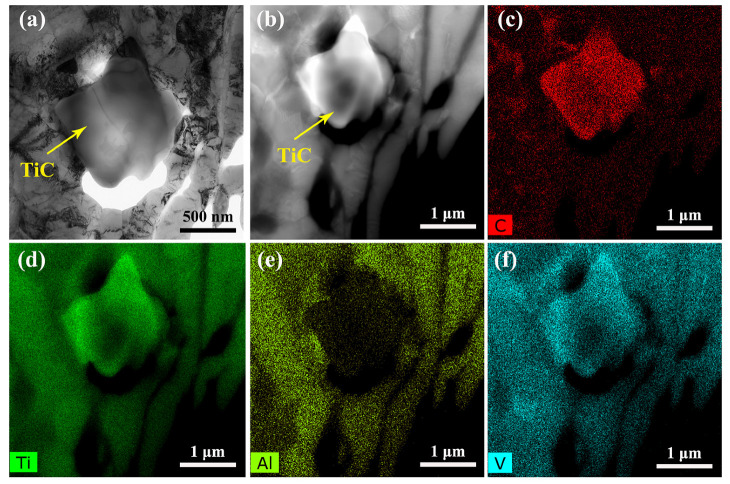
TEM images of TiC/Ti64 composites with melt hydrogenation compressed at 900 °C/0.01 s^−1^: (**a**) bright-field image, (**b**) HAADF image, and EDS mapping results for (**c**) C, (**d**) Ti, (**e**) Al, and (**f**) V.

Figure 5 shows a bright-field image, high-angle annular dark-field image (HAADF), and EDS mapping results of deformed TiC/Ti64 composites at 900 °C and 0.01 s^−1^ with melt hydrogenation. According to Figure 5a,b, the TiC phase has a near-equiaxial morphology, which is different when compared with the morphology shown in Figure 1 and Figure 2. The EDS mapping results shown in Figure 5c–f indicate that the phase shown in Figure 5a,b is enriched with C element, which further confirms this phase is TiC. After hot compression, the feather-like or dendritic TiC particles fragment into smaller pieces, which is caused by the force from neighboring grains during high-temperature deformation.

**Figure 6:**
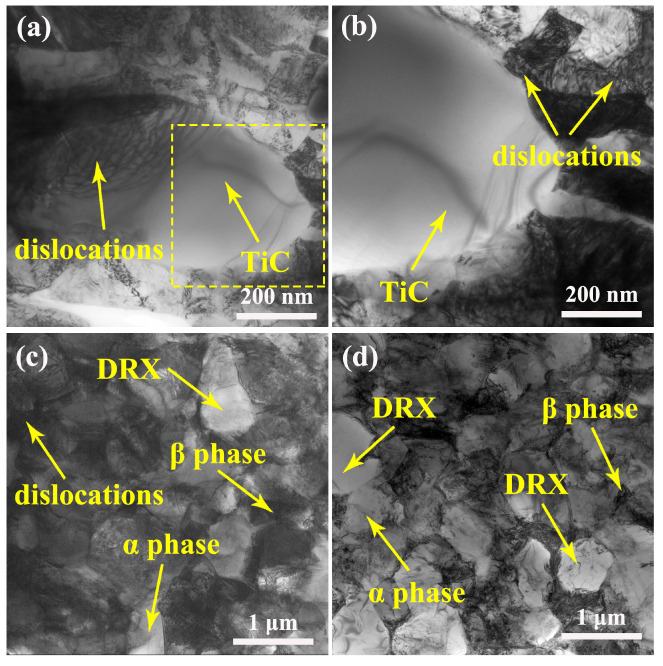
Bright-field images of TiC and Ti64 matrices in composites after hot compression at 900 °C/0.01 s^−1^: (**a**–**c**) without melt hydrogenation, (**d**) with melt hydrogenation.

TEM bright-field images of deformed TiC/Ti64 composites at 900 °C/0.01 s^−1^ without hydrogenation are shown in Figure 6. As shown in Figure 6a, there is a large number of dislocations in the TiC phase, which is caused by plastic deformation during hot compression. When the dislocations accumulate to form piles, there will be cracks generating inside the TiC phase, and then the original TiC phase forming during solidification will fragment into smaller pieces. An enlarged image of the yellow square in Figure 6a is shown in Figure 6b. The TiC phases are the obstacles for dislocations slipping, and there are many dislocations stacking at the interface between TiC and the matrix, which will increase the flowing stress of composites. Figure 6c shows the microstructure of the Ti64 matrix, where it can be seen that there is a high density of dislocations, and the volume fraction of DRX grains is low. DRX is the main mechanism to consume the dislocations, and if the proportion of DRX is low, the dislocations and the work-hardening effect cannot be balanced out, which will induce higher peak stress. In comparison, melt hydrogenation significantly accelerates the DRX process, as shown in Figure 6d, and the density of dislocations is also decreased.

## 4. Discussion

### 4.1. Effect of Melt Hydrogenation on Distribution and Morphology of TiC

Based on the Ti-C binary phase diagram [4], the C content in this study belongs to the hypoeutectic composition, meaning the TiC formed in this study is in the eutectic TiC phase. During solidification, the primary β grains nucleate first, and the growth and distribution of eutectic TiC phases depend on the morphology of primary β grains. When there is a large number of primary β grains, only a small amount of residual melt exists between the dendrites, and the eutectic β grains can attach to the primary β grains, which induces eutectic TiC phases to form between the dendrites’ channels and then become strip or feather-like particles [16,17]. In comparison, when there is a large amount of residual melt between the dendrites, the eutectic β grains not only grow on the primary β grains but also grow with TiC cooperatively. In this study, the TiC phases were distributed homogenously in the composites without melt hydrogenation, as shown in Figure 1a and Figure 2a, because the primary β grains had a lower growth rate and there was a large amount of residual melt between the dendrites. In comparison, the TiC phases were distributed in the narrow dendritic channels of composites with melt hydrogenation, as shown in Figure 1c and Figure 2d, because melt hydrogenation promoted the growth of primary β grains by accelerating atomic diffusion [18]. As a result, most TiC was distributed along the primary β grain boundaries in TiC/Ti64 composites with melt hydrogenation.

In addition, TiC in composites with melt hydrogenation also had large sizes, as shown in Figure 1c and Figure 2d. TiC had a NaCl-type cubic crystal structure, in which the Ti atoms occupied all positions in the face-centered cube, and C atoms occupied all octahedral gaps in the Ti cubic configuration [19]. TiC had cubes or minimal octahedral structure units with strong covalent bonds, which could not be broken at high temperatures due to their high binding energy. Therefore, TiC existed in large-sized clusters in the melt before solidification. Once the temperature becomes lower than the forming temperature of TiC phases, they will grow directly through atom-packing in the growth steps [20], meaning the atomic packing rate has a critical influence on their growth rate. TiC has a symmetrical crystal structure, and the growth rate and trend in all directions are also completely symmetrical, so there is no preferred growth direction for TiC. It has been reported that melt hydrogenation induces overheating on the melt surface [18], and hydrogen also can accelerate atomic diffusion. Therefore, the TiC phases in composites with melt hydrogenation have larger sizes compared to those without melt hydrogenation, but their shape is not obviously changed, as shown in Figure 2.

### 4.2. Effect of Melt Hydrogenation on Hot Workability of TiC/Ti64 Composites

During hot compression, the original as-cast grains become deformed structures, and the eutectic TiC particles become smaller broken pieces. The hot workability of TiC/Ti64 composites is decided by the atomic bonding force, phase content, dislocation density, and DRX [21]. As shown in Figure 3, melt hydrogenation can significantly decrease the peak flowing stress and then improve the hot workability. Considering the volume fraction of TiC is only 2.5%, the high-temperature performance of the titanium matrix had a dominant role in shaping the hot workability of TiC/Ti64 composites in this study. Hydrogen atoms can decrease the atomic bonding force in titanium alloys [22]; therefore, hydrogen-induced reduction in the atomic bonding force of the Ti-6Al-4V matrix plays a critical role in decreasing the flowing stress of TiC/Ti64 composites. Ti64 matrix alloy is a dual-phase titanium alloy, which is composed of the hcp-α phase and the bcc-β phase [23]. The bcc-β phase has more slipping systems compared to the hcp-α phase [24], so a higher content of the ductile β phase can decrease the peak flowing stress of TiC/Ti64 composites during hot processing. As shown in Table 1, melt hydrogenation decreases the β/(α + β) phase transition temperature from 1024 ± 9 to 998 ± 7 °C because hydrogen is a β-stabilizing element. The compressing temperatures in this study (700, 800, and 900 °C) were all in the (α + β) phase region, so melt hydrogenation decreased the peak stress of TiC/Ti64 composites because hydrogen acted as a β-stabilizing element that improved the ductile β phase content in the (α + β) phase region.

The flowing stress of TiC/Ti64 composites is also decided by the high-temperature microstructure evolution. During hot compression, the work-hardening effect is dominant before the peak stress, whereas the DRX-induced softening effect is dominant after the peak stress. In order to reduce the peak stress, a high volume fraction of DRX grains is required as the DRX can efficiently compensate for the work-hardening effect, and dislocation slipping is also crucial to high-temperature deformation behaviors. As shown in Figure 4b,e, melt hydrogenation promotes the proportion of DRX to increase from ~62.7% to ~83.2%, and the TEM results shown in Figure 6 confirm this finding. The DRX in the titanium matrix composites can be considered discontinuous DRX, which is completed by nucleation and growth of newly formed grains [25]. During the growth of DRX grains (or the migration of DRX grain boundaries), a large number of dislocations will be consumed [26]. When the DRX-induced softening effect is stronger than the work-hardening effect, the flowing stress will reach the peak value and then decrease continuously. The migration of DRX grain boundaries is mainly controlled by atomic diffusion, and hydrogen atoms have a high diffusion coefficient in a titanium alloy [27], which can accelerate the diffusion of solute atoms. Therefore, melt hydrogenation accelerates atomic diffusion and then increases the migration of DRX grain boundaries, which will improve the proportion of DRX grains.

Furthermore, as discussed earlier, the formation of DRX grains is closely related to dislocation slipping, and higher mobility of dislocations cannot only accelerate the DRX but also has benefits for the plastic deformation at high temperatures. Different from the Ti-6Al-4V matrix alloy, the addition of TiC particles introduces a large number of obstacles for dislocation slipping in composites, which will make dislocation slipping or climbing harder. If the dislocations have low mobility, there will be a large number of dislocations accumulating at the interface between TiC particles and the matrix alloy, which will increase the flowing stress. It has been reported that the mobility of dislocations has been improved by hydrogen atoms in titanium alloys [9,28]. Higher mobility of dislocations accelerates the process of DRX and decreases the number density of dislocations, which decreases the peak flowing stress. In brief, the enhanced hot workability of hydrogenated TiC/Ti64 composites results from hydrogen atoms decreasing the atomic bonding force, hydrogen reducing the β/(α + β) transition temperature, a higher proportion of DRX, and higher mobility of dislocations.

## 5. Conclusions

This study employed melt hydrogenation to fabricate TiC/Ti-6Al-4V composites, and the effects of melt hydrogenation on the morphology and distribution of TiC particles, hot compression behaviors, and mechanisms for improvement of the hot workability were analyzed. The main conclusions are as follows.

(1) Melt hydrogenation did not change the shape of the eutectic TiC phase, but the size of eutectic TiC and the primary β phase were significantly increased. As a result, many large-sized eutectic TiC were distributed along the grain boundaries of primary β grains;
(2) The peak flowing stress of TiC/Ti64 composites in the (α + β) phase region was reduced by melt hydrogenation, most significantly with a strain rate of 0.01 s^−1^. When compressed at 900 °C/0.01 s^−1^, the peak stress decreased from 241 ± 9 to 190 ± 8 MPa (decreased by ~51 MPa and 21.2%), and the best improvement of hot workability was obtained;
(3) The decreased atomic bonding force, reduced β/(α + β) transition temperature, higher proportion of DRX, and higher mobility of dislocations by hydrogen atoms all contributed to the enhanced hot workability of TiC/Ti64 composites.

## Figures and Tables

**Table 1:** Hydrogen content and β/(α + β) transition temperature of TiC/Ti64 composites.

NO.	Hydrogen Percentagein Gas Mixture (%)	Hydrogen Content (wt.%)	β/(α + β) Transition Temperature (°C)
unhydrogenated	0	0	1024 ± 9
hydrogenated	20	0.0503 ± 0.0027	998 ± 7

## Data Availability

Some or all data, models, or code that support the findings of this study are available from the corresponding author on reasonable request.

## References

[1] Zhou Y., Yang F., Chen C., Shao Y., Lu B., Sui Y., Guo Z. (2022). Mechanical property and microstructure of in-situ TiB/Ti composites via vacuum sintering and hot rolling. J. Alloys Compd..

[2] Xiaolong C., Zulei L., Yanhua G., Zhonggang S., Yaoqi W., Lian Z. (2022). A study on the grain refinement mechanism of Ti-6Al-4V alloy produced by wire arc additive manufacturing using hydrogenation treatment processes. J. Alloys Compd..

[3] An Q., Wang S., Huang L., Wang C., Qian Q., Jiang Y., Wang L., Geng L. (2022). Experimental and first-principles study on TiB/TiC interface in hybrid (TiB + TiC)/Ti6Al4V composite. Ceram. Int..

[4] Ya B., Zhou B., Yang H., Huang B., Jia F., Zhang X. (2015). Microstructure and mechanical properties of in situ casting TiC/Ti6Al4V composites through adding multi-walled carbon nanotubes. J. Alloys Compd..

[5] Han C., Babicheva R., Chua J.D.Q., Ramamurty U., Tor S.B., Sun C.-N., Zhou K. (2020). Microstructure and mechanical properties of (TiB + TiC)/Ti composites fabricated in situ via selective laser melting of Ti and B4C powders. Addit. Manuf..

[6] Wei Z.J., Cao L., Wang H.W., Zou C.M. (2011). Microstructure and mechanical properties of TiC/Ti-6Al-4V composites processed by in situ casting route. Mater. Sci. Tech. Ser..

[7] Wang X., Wang L., Luo L., Yan H., Li X., Chen R., Su Y., Guo J., Fu H. (2017). High temperature deformation behavior of melt hydrogenated (TiB + TiC)/Ti-6Al-4V composites. Mater. Des..

[8] Wang X., Wang L., Luo L., Liu X., Tang Y., Li X., Chen R., Su Y., Guo J., Fu H. (2017). Hot deformation behavior and dynamic recrystallization of melt hydrogenated Ti-6Al-4V alloy. J. Alloys Compd..

[9] Zhao J.W., Ding H., Hou H.L., Li Z.Q. (2010). Influence of hydrogen content on hot deformation behavior and microstructural evolution of Ti600 alloy. J. Alloys Compd..

[10] Wang X., Wang L., Luo L., Xu Y., Li X., Chen R., Su Y., Guo J., Fu H. (2017). Hydrogen induced softening and hardening for hot workability of (TiB + TiC)/Ti-6Al-4V composites. Int. J. Hydrogen Energy.

[11] Wang X., Wang L., Luo L., Liu X., Tang Y., Yan H., Yao L., Su Y., Guo J., Fu H. (2018). Positive effect of hydrogen on interface of in situ synthesized Ti-6Al-4V matrix composites. Mater. Sci. Eng. A.

[12] Wang L., Zhang L., Luo L., Wang B., Yan H., Chen R., Su Y., Guo J., Fu H. (2020). Effect of melt hydrogenation on microstructure evolution and tensile properties of (TiB + TiC)/Ti-6Al-4V composites. J. Mater. Res. Tech..

[13] Lin X., Dong F., Zhang Y., Yuan X., Huang H., Zheng B., Wang L., Wang X., Luo L., Su Y. (2019). Hot-deformation behaviour and hot-processing map of melt-hydrogenated Ti 6Al 4V/(TiB+TiC). Int. J. Hydrogen Energy.

[14] Bai M., Namus R., Xu Y., Guan D., Rainforth M.W., Inkson B.J. (2019). In-situ Ti-6Al-4V/TiC composites synthesized by reactive spark plasma sintering: Processing, microstructure, and dry sliding wear behaviour. Wear.

[15] Wang X., Wang L., Yang F., Luo L., Yan H., Liu X., Li X., Chen R., Su Y., Guo J. (2018). Hydrogen induced microstructure evolution of titanium matrix composites. Int. J. Hydrogen Energy.

[16] Peterson J., Issariyapat A., Umeda J., Kondoh K. (2022). The effects of heat treatment and carbon content on the microstructure and mechanical properties of laser powder bed fusion Ti-6Al-4V with dissolved TiC particles. J. Alloys Compd..

[17] Ochonogor O.F., Meacock C., Abdulwahab M., Pityana S., Popoola A.P.I. (2012). Effects of Ti and TiC ceramic powder on laser-cladded Ti–6Al–4V in situ intermetallic composite. Appl. Surf. Sci..

[18] Oh J.-M., Roh K.-M., Lim J.-W. (2016). Brief review of removal effect of hydrogen-plasma arc melting on refining of pure titanium and titanium alloys. Int. J. Hydrogen Energy.

[19] Wang X., Li S., Han Y., Huang G., Mao J., Lu W. (2022). Visual assessment of special rod-like α-Ti precipitates within the in situ TiC crystals and the mechanical responses of titanium matrix composites. Compos. Part B Eng..

[20] Zhang Y., Sun J., Vilar R. (2011). Characterization of (TiB+TiC)/TC4 in situ titanium matrix composites prepared by laser direct deposition. J. Mater. Process Tech..

[21] Senkov O.N., Froes F.H. (1999). Thermohydrogen processing of titanium alloys. Int. J. Hydrogen Energy.

[22] Teter D.F., Robertson I.M., Birnbaum H.K. (2001). The effects of hydrogen on the deformation and fracture of β-titanium. Acta Mater..

[23] Shen J., Kotha S., Noraas R., Venkatesh V., Ghosh S. (2022). Developing parametrically upscaled constitutive and crack nucleation models for the α/β Ti64 alloy. Int. J. Plast..

[24] Shen J., Sun Y., Ning Y., Yu H., Yao Z., Hu L. (2019). Superplasticity induced by the competitive DRX between BCC beta and HCP alpha in Ti-4Al-3V-2Mo-2Fe alloy. Mater. Charact..

[25] Arun Babu A.K., Mozumder Y.H., Saha B.R., Subramanya Sarma C.V., Mandal A.S. (2018). Hot-workability of super-304H exhibiting continuous to discontinuous dynamic recrystallization transition. Mater. Sci. Eng. A.

[26] Shen J., Zhang L., Hu L., Sun Y., Gao F., Liu W., Yu H. (2021). Effect of subgrain and the associated DRX behaviour on the texture modification of Mg-6.63Zn-0.56Zr alloy during hot tensile deformation. Mater. Sci. Eng. A.

[27] Chen Y.Z., Barth H.P., Deutges M., Borchers C., Liu F., Kirchheim R. (2013). Increase in dislocation density in cold-deformed Pd using H as a temporary alloying addition. Scr. Mater..

[28] Xu Y., Zhang B. (2021). Effects of hydrogen as a solid solution element on the deformation behavior of a near-alpha titanium alloy. Mater. Sci. Eng. A.

